# Current topics of interdisciplinary cooperation between engineering and human sciences

**DOI:** 10.1007/s41449-023-00352-y

**Published:** 2023-02-01

**Authors:** Angela Dressler, Nina Gerber, Angela Menig, Otilia Pasnicu, Alina Stöver, Joachim Vogt

**Affiliations:** Work and Engineering Psychology Research Group, Technical University of Darmstadt, Alexanderstr. 10, 64283 Darmstadt, Germany

**Keywords:** Technikpsychologie, Engineering Psychology, Human-Machine-Interaction, Technikpsychologie, Ingenieurpsychologie, Mensch-Maschine-Interaktion

## Abstract

In this article, we highlight current research directions in the Technikpsychologie research area, using the example of the interdisciplinary research work of FAI (Work and Engineering Psychology Research Group at the Technical University of Darmstadt) and the articles included in this special issue. To this end, we relate the articles in this special issue from the research areas of road traffic planning (Hupfer et al.), usable IT security and privacy solutions (Renaud), social aspects of technically mediated communication (Diefenbach), human-centered interface design (Mucha et al.), aviation safety (Santel), human-centered design of autonomous vehicles (Lindner & Stoll), and perceptual psychology-oriented product design (Zandi & Khanh) to current research projects at FAI.

*Practical Relevance*

Technical products only offer added value by efficiently supporting users in achieving their goals if they have been developed appropriately for the context of use and the individual characteristics of the users. The human-centered design of—especially technical—products reflects this through an iterative and participatory development process. In this article, we describe nine examples of such human-centered design of technology products. The research results and the methods presented provide insights for developers and decision-makers in the fields of transportation, IT, vehicle development and general product design.

## Abstract (de)

In diesem Artikel zeigen wir aktuelle Forschungsrichtungen der Technikpsychologie am Beispiel der interdisziplinär geprägten Forschungsarbeit der FAI (Forschungsgruppe für Arbeits- und Ingenieurpsychologie an der Technischen Universität Darmstadt) und den in diesem Themenheft enthaltenen Artikeln auf. Zu diesem Zweck setzen wir die Artikel dieses Themenheftes aus den Forschungsbereichen Verkehrsplanung (Hupfer et al.), nutzbare IT Sicherheit- und Privatsphärelösungen (Renaud), soziale Aspekte der technisch mediierten Kommunikation (Diefenbach), menschzentrierte Schnittstellengestaltung (Mucha et al.), Flugsicherheit (Santel), menschzentrierte Gestaltung von autonomen Fahrzeugen (Lindner & Stoll) sowie wahrnehmungspsychologisch orientierte Produktgestaltung (Zandi & Khanh) zu aktuellen Forschungsprojekten der FAI in Verbindung.

*Praktische Relevanz*

Technische Produkte bieten nur dann einen Mehrwert, wenn sie Nutzende effizient in der Erreichung ihrer Ziele unterstützen, wenn diese passend für den Nutzungskontext und die individuellen Charakteristiken der Nutzenden entwickelt wurden. Die menschzentrierte Gestaltung von – insbesondere technischen – Produkten bildet dies durch den iterativ und partizipativ geprägten Entwicklungsprozess ab. In diesem Artikel beschreiben wir insgesamt neun Beispiele für eine solche menschzentrierte Gestaltung von Technikprodukten. Die Forschungsergebnisse ebenso wie die vorgestellten Methoden liefern hierbei Erkenntnisse für Entwickler:innen und Entscheider:innen aus den Bereichen Verkehr, IT, Fahrzeugentwicklung sowie genereller Produktgestaltung.

At FAI, we pursue several research streams of Technikpsychologie. We have included examples for these different research areas in this special issue, with all articles describing recent research studies or summarizing the literature in a research field by an expert for this application area. These experts may be psychologists, but also researchers from the computer sciences, design, or engineering. In this article, we provide information on the research we conduct in the specific research areas and how this relates to the articles included in this special issue.

The ideas of Vogt et al. (described in the first article of this special issue) are picked up and further elaborated by Kurt Landau. In addition to the engineering psychology perspective, work-scientific and productivity related issues are discussed. Thus, a broader scope of history and disciplines is provided. Professor Dr.-Ing. Kurt Landau worked as systems analyst in France and Switzerland. He was Director of the Ergonomics Department and Foreign Affairs of the German REFA (Time and Motion Studies). This was followed by 12 years as Professor of Ergonomics at Stuttgart University. He then was the head of the Institute of Ergonomics at the Technical University of Darmstadt from 1995 to 2006. Professor Landau was President of the German Ergonomics Society and editor-in-chief of the Zeitschrift für Arbeitswissenschaft. He has received several awards and honours, such as the honour medal of the German Ergonomics Society or the IEA-Fellowship award. In 2016 he was distinguished as Honorary Member of the German Ergonomics Society. Since his retirement he is Professor at the École de Téchnologie Supérieure (ETS) Montréal, Canada. He has authored more than 450 research articles and books.

## Road traffic planning

A first application area of engineering psychology is covered by Hupfer et al. They describe state of the art safety and certainty of road design and how it can be improved by social psychology. Especially decision-making in this area, often done by lay-people in communal committees, will benefit from psychological theories and data. FAI is currently conducting extensive trials with bicycles, especially pedelecs, in cooperation with Hupfer and colleagues. Our test people found, for example, many dangerous transitions from bicycle paths, roads, sidewalks, and traffic lights. Too small bicycle lanes, high curbs and damaged asphalt can be found all over. This may sound trivial; however, it must constantly be put to mind of decision-makers when new roads are planned. Moreover, the basic building blocks of the transport system have changed (Fig. 1 of Hupfer et al.). The vehicles, in this case bicycles, have changed to the faster and heavier, so that state of the art is not sufficient in the future. Finally, also society is changing towards climate friendly bicycle traffic requirements. In our view and in the light of our ongoing bicycle study, the design of biking facilities leads to a safe situation for all users and encourage for cycling. The space for bicycles must avoid minimum spaces and missing separations like depicted in Fig. 2 of Hupfer et al. Prof. Dr.-Ing. Christoph Hupfer studied Civil Engineering in Kaiserslautern, Germany. He specialized in Traffic Engineering including facility design and capacity as well as in Transportation Planning focusing on transformation towards sustainable mobility. Traffic safety was the area of his doctoral thesis. Since 2001 he is Professor for Traffic Planning and Traffic Technology as well as Head of the Baden-Württemberg Institute for Sustainable Mobility.

**Fig. 1:**
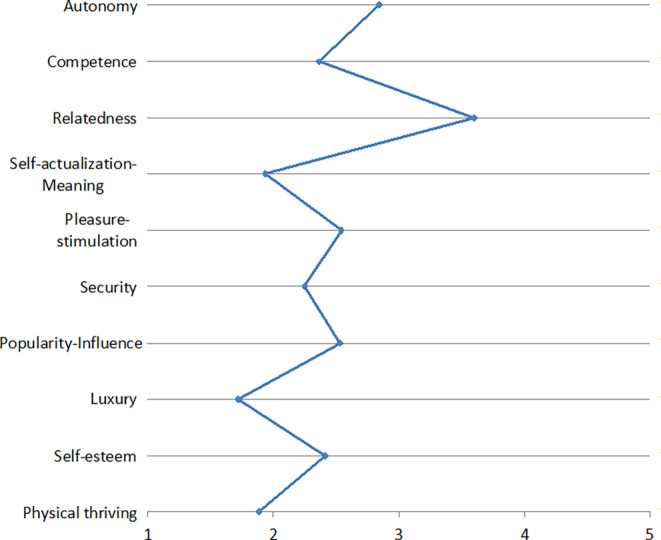
Needs associated with using messengers Bedürfnisse, die mit der Nutzung von Messenger-Diensten verbunden werden

**Fig. 2:**
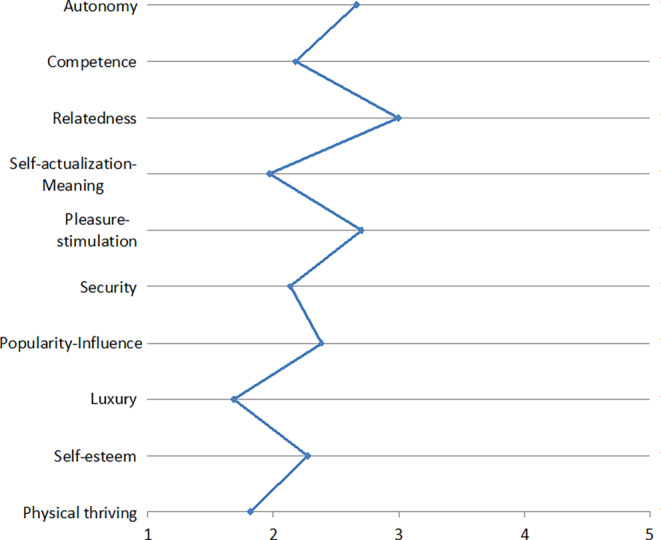
Needs associated with using Online Social Networks Bedürfnisse, die mit der Nutzung von sozialen Netzwerken verbunden werden

## Usable security & privacy

Karen Renaud discusses another stream of Technikpsychologie research in her article: the field of usable security and privacy. Studies in this research area are frequently conducted at the intersection of computer science and psychology. Still, other disciplines such as the legal sciences or design might also be involved.

Karen Renaud is a Scottish computing scientist at the University of Strathclyde in Glasgow, working on all aspects of human-centered security and privacy. She was educated at the Universities of Pretoria, South Africa, and Glasgow. Her research has been funded by the Association of Commonwealth Universities, the Royal Society, the Royal Academy of Engineers and the Fulbright Commission. She is particularly interested in deploying behavioural science techniques to improve security behaviours, and in encouraging end-user privacy-preserving behaviours. Her research approach is multi-disciplinary, essentially learning from other, more established, fields and harnessing methods and techniques from other disciplines to understand and influence cybersecurity behaviours.

In recent years, the FAI has worked on multiple research projects dealing with the human factor aspects of cybersecurity. The expertise available here is reflected, for example, in FAI’s membership in the National Research Center for Applied Cybersecurity ATHENE (ATHENE Center 2022) and the Research Training Group RTG Privacy and Trust for Mobile Users (Technische Universität Darmstadt 2022).

The research focus in these projects has often been on end users, who, for example, should be supported in:

- Using *secure authentication methods* (Zimmermann et al. 2022; Zimmermann and Gerber 2020a; Marky et al. 2020a; Zimmermann et al. 2019b; Renaud and Zimmermann 2019; Mayer et al. 2019; Zimmermann et al. 2018b; Renaud and Zimmermann 2018a, b; Marky et al. 2018; Renaud et al. 2017; Zimmermann et al. 2017a; Gerber and Zimmermann 2017; Zimmermann and Gerber 2017; Renaud and Zimmermann 2017)
- Using *end-to-end encryption (E2EE)* for their digital communications (Brendel and Gerber 2019; Gerber et al. 2018c, d; Ghiglieri et al. 2018; Zimmermann et al. 2017a, b)
- Making informed decisions regarding the handling of their* private data* (Marky et al. 2022; Gerber et al. 2021; Stöver et al. 2021; Balthasar et al. 2021; Schürmann et al. 2020; Marky et al. 2020b, c; Kulyk et al. 2020a, b; Gerber et al. 2019a, b; Zimmermann et al. 2019a, c; Gerber et al. 2018a, b; Karegar et al. 2018; Zimmermann et al. 2018a; Kulyk et al. 2018a, b)

For example, in a lab study in which 41 participants interacted with twelve different authentication schemes chosen based on an objective rating scheme (Bonneau et al. 2012; Zimmermann et al. 2019b, 2018b), we found that the classic password, followed directly by fingerprint, was rated best by users in terms of preference, usability, and intention to use, and users expected the fewest problems and effort with this scheme (Zimmermann and Gerber 2020b). Further research, consequently focusing on the password as most favoured authentication scheme, revealed that hybrid password meters, i.e., password meters that include password feedback, a feedback nudge, and additional guidance for creating a secure password, enable users to create better passwords compared to each intervention (feedback, nudge, additional guidance) on its own (Zimmermann et al. 2022). More specifically, Zimmermann et al. (2022) found differences in terms of password strength, memorability, and user perceptions applying a between-subject design in an online study with 646 participants who used one out of nine variations of password meters. This effect, however, seems to be independent of the type of feedback nudge used. In another online study following a 2 × 2 × 4-within and between subject design, Zimmermann and Renaud (2021) found that hybrid nudges, i.e., nudges that provide additional information, lead to better security decisions, including password choice, compared to providing mere information. Renaud and Zimmermann (2018a) further provided guidelines for conducting nudge experiments, particularly in the password authentication context. Other studies explored innovative authentication methods, e.g., relying on 3D-printed items for two-factor authentication (Marky et al. 2020a), or offering shoulder-resistant text password entry on gamepads (Mayer et al. 2019).

Another set of studies focused on how users perceive end-to-end-encryption (E2EE). For this purpose, we conducted interviews with twenty WhatsApp users following the introduction of E2EE in WhatsApp in 2016 (Gerber et al. 2018c, d). We identified several misconceptions, e.g., some participants thought that encrypted messages would be transmitted directly between smartphones, omitting the server as an intermediate step, while others thought that they had to enter a password to en- and decrypt the messages send via E2EE. Still, many participants correctly identified the sender and recipient as the weakest link in end-to-end-encrypted messages. Our results further indicate that most users do not trust WhatsApp; this is reflected in the finding that about half of our participants thought that even with E2EE, their messages could still be eavesdropped by WhatsApp’s employees and cooperation partners, as well as hackers and governmental institutions. In Ghiglieri et al. (2018), we describe the results of a cognitive walkthrough conducted by experts and lay users to explore, whether the E2EE tools PGP and S/MIME can be applied by non-expert users if they have already been set up by an expert user in Mozilla Thunderbird to enable email E2EE. We identify several suggestions for improvement, indicating that usability-wise email E2EE is not yet matured.

Regarding usable privacy, we have investigated in multiple contexts how users’ privacy behaviours can be explained and how they can be supported in making informed decisions about the handling of their private data. For example, in Gerber et al. (2018a) we aimed to investigate which factors influence privacy attitude, intention, and behaviour. For this purpose, we conducted a systematic literature review and synthesized the effect sizes found in user studies that used regression analyses or structural equation modelling. Thus, we approach an explanation for the so-called privacy paradox, which describes the phenomenon that users frequently state that they attach great importance to protecting their privacy, but this attitude is usually not reflected in their behaviour (Gerber et al. 2017c). Based on the data available, we conclude that the benefits of disclosing data and the perceived risk seem to play a major role in the decision for or against disclosing private data, while demographic or personality factors may be negligible. Several studies focused on privacy protection in Internet of Things (IoT)-equipped environments: In a mixed-method approach, we derive a design space for measures aimed to protect the privacy of guests in households which own IoT devices from insights gained in in-depth interviews with 21 IoT device owners and a survey study with 264 participants (IoT device owners and non-owners). We found that, e.g., guests value feedback about the status of their privacy protection, while IoT device owners fear that such feedback could compromise the aesthetics of their living spaces (Marky et al. 2022). We further proposed an interface-based prototype for controlling IoT-equipped households using local information processing, thus protecting the IoT owner from surveillance and hacking attacks (Gerber et al. 2021). This prototype was developed following a user-centered-design process, i.e., it was refined usability-wise in an iterative process based on several user evaluation studies. An innovation compared to existing systems is that all IoT devices can be controlled task-based, for example, to create a morning routine, while traditional systems are mostly controlled device-based. Further studies aimed, for example, to identify users’ mental models of smart homes (Zimmermann et al. 2019a), or privacy threats in smart environments (Gerber et al. 2018b, 2019a; Zimmermann et al. 2019c). Other experimental lab and online studies explored, for example, how users can be supported in selecting privacy-preserving mobile apps (Stöver et al. 2021; Gerber et al. 2017a, b; Kulyk et al. 2019), deciding about using Single Sign On (SSO) login (Karegar et al. 2018), or how they perceive and interact with cookie consent notices (Kulyk et al. 2020a, 2018a, b).

### Psychological needs

In her article, the author Karen Renaud considers the psychological needs of expert users, e.g., software engineers. We explored the needs of users in several studies: For example, in Gerber et al. (2017b), we describe a survey study (*N* = 280) in which we investigated the influence of psychological needs on the implementation of privacy protection measures, including the management of privacy settings, that users applied on Facebook. Using regression analyses, we found that primarily participants who used Facebook in order to feel meaningful and stimulated applied lax privacy settings. We did not find a statistically significant relationship between usage motivation (i.e., psychological needs that the participants aim to fulfil by using Facebook) and the deployment of other protection strategies besides managing privacy settings, such as blocking contacts or deleting posts or photo/video tags. However, our results suggest that especially Facebook users scoring high on extraversion and low on agreeableness tend to use such protection strategies.

In another study described by Zimmermann and Gerber (2020a), we asked 17 participants in semi-structured interviews including a card-sorting task about their use of various digital applications and how these relate to the psychological needs identified by Sheldon et al. (2001). Using open coding for the analysis, we identified four psychological needs that our participants strive to fulfill by using all the applications considered in our study (i.e., messengers, social networks, cloud services, digital assistants, and smart TVs). These four psychological needs may thus be relevant for users independent of the specific use context: (1) relatedness-belongingness, (2) competence-effectance, (3) pleasure-stimulation, and (4) autonomy-independence. We further found that the importance of four additional psychological needs seem to be application-specific: (5) security (for cloud services and messengers), (6) popularity-influence (for social networks), (7) self-actualization-meaning (for social networks), and (8) money-luxury (for smart TVs). Our findings are largely consistent with previous studies that have examined the relevance of psychological needs based on self-determination theory (Ryan and Deci 2000), which postulates the three psychological needs autonomy, competence, and relatedness as the basis for self-motivation and personality integration. In her article, Karen Renaud also refers to these three psychological needs. Our results suggest that these three needs are indeed application-independently of great importance for users of digital products and services. However, we add a fourth component to this set of needs: pleasure-stimulation, i.e., feeling well entertained and enjoying what one is doing.

Currently, we are working on a set of studies that investigate what psychological needs are relevant for users of a mobile privacy assistant (manuscripts in preparation; Stöver et al. 2023b, 2020).

### Expert users

Karen Renaud also considers end users in her article, but primarily focuses on software engineers, who belong to the group of expert users. A main conclusion of Renaud’s article is that software developers need to be supported in their efforts to develop secure software. We took a different angle in a recent study, in which we aimed to shine light on how another group of expert users, i.e., website owners, should be supported in providing secure and privacy-preserving website for their visitors (Stöver et al. 2022, 2023a; Maaß et al. 2021). We conducted, for example, an analysis of widely used content management systems (CMPs) that can be used by website owners to create cookie consent notices (Stöver et al. 2022). For this purpose, two researchers independently created cookie consent notices using these CMPs a) with the default settings, b) with a design that is as neutral as possible, i.e., it should be equally easy to accept and decline the use of cookies, c) with a so-called bright pattern, which should encourage users to decline the use of cookies. Two additional researchers then coded screenshots of the cookie consent notices generated in the prior step according to the dark pattern taxonomy proposed by Gray et al. (2018). The goal here was to analyse whether the generated cookie consent notices contained dark patterns, i.e., deceptive design elements that aim to nudge users towards accepting the use of cookies, such as highlighting certain options visually (dark pattern: interface interference) or hiding the “decline” button on the second layer of the cookie consent notice (obstruction) (see also Soe et al. 2020). The researchers who conducted the coding were not aware which cookie consent notices were generated with the aim to employ a neutral design, bright patterns, or relied on default settings. Our results indicate that cookie consent notices created with the default settings of widely spread CMPs frequently contained dark patterns; 60% of the cookie consent notices were found to employ at least one dark pattern design element. We further found that some CMPs do not even offer the possibility to create neutral or “bright” cookie consent notices. Of all CMPs considered, 37.5% were found to still include dark patterns, even if the notices were created with the aim of being neutral or highlighting the privacy-preserving option (Stöver et al. 2022). Our findings underline the importance of bringing expert users such as developers and website owners into the focus of usable security and privacy research and exploring how they can be supported in making the products they develop and operate more secure and privacy-friendly for end users.

Karen Renaud further refers to a common stereotype of software engineers, who are frequently considered as being anti-social. A recent FAI study touched on this topic (Gerber and Marky 2022) by exploring how people who are well-versed in cybersecurity and privacy topics can apply their knowledge to support their peers in IT security and privacy matters. In 13 in-depth interviews and three co-creation workshops with 11 Security and Privacy adepts (S&P adepts), we found that S&P adepts aim to educate people from their social environment but focus on such people with whom they have a close social relationship. This might be because a trusting relationship is essential for S&P adepts to address what they consider to be a potentially sensitive topic. Further, although most S&P adepts in our study reported to enjoy being asked for advice, they only talk proactively to other S&P experts about topics related to security and privacy, since they assume less S&P-savvy people are not interested in these topics, but also due to the fear of criticizing others and being preachy. This is further complicated by the fact that the S&P adepts in our studies reported to not feel in the moral position to judge others’ security and privacy behaviour, e.g., because they frequently differ from what they would consider an ideal secure and privacy-preserving behaviour themselves. We hence conclude that we need conversation starters that trigger a situation in which lay users ask S&P adepts directly for advice. Such a conversation starter could, for example, be the official delegation of security and privacy in the private context, following the lines of data protection officers or IT security commissioners in the corporate context.

Additionally, Karen Renaud, Verena Zimmermann, Alexandra von Preuschen (Justus-Liebig-Universität Gießen) and Nina Gerber are currently working on a set of studies that aims to explore how IT Security professionals are perceived by lay users.

### Human-as-a-solution

In a somewhat earlier project, we looked at the corporate context and investigated which factors in companies lead to employees behaving in a security-compliant manner (Mayer et al. 2017; Gerber et al. 2016). In a first step, we conducted a survey study with 200 German employees recruited via a panel. Using regression analysis, we found that employees who work in companies that reward productivity goal achievement tend to report being less security-compliant compared to employees in whose companies’ productivity goal achievement is not rewarded; indicating negative effects of conflicting goals (Gerber et al. 2016; Mayer et al. 2017). Based on these results, we developed a concept for setting information security goals in organizations building on actionable behavioural recommendations from information security awareness materials. The evaluation of this goal setting concepts with 90 employees in three small-to medium-sized organizations (SMEs) showed that 1) information security awareness materials should include actionable behavioural recommendations, and 2) employees should be able to select multiple pre-defined goals as well as specify their own goals (Mayer et al. 2017). Our results call attention to the fact that users, not exclusively but especially in a professional context, often have to deal with conflicting goals. Therefore, in our view, it is not appropriate to consider users as the weakest link in the defense of IT security, but rather to target the systematic level and investigate how to effectively support users in their efforts to improve IT security.

In her article, Karen Renaud also calls for a shift from a human-as-a-problem to a human-as-a-solution perspective. Karen Renaud has pursued this approach together with the then FAI staff member Verena Zimmermann (Zimmermann and Renaud 2019; Renaud et al. 2021), who has been an assistant professor at ETH Zurich since August 2022 and continues to work with FAI in various collaborative projects.

## Social aspects of digitally mediated communication

In her article, Sarah Diefenbach reports on the results of a mixed-method study exploring the social interactions of co-workers in a teleworking set up from a psychological perspective. She identified several communication conflicts, including the lack of behavioural rules and social feedback, and norm violations.

Sarah Diefenbach is professor for market and consumer psychology at the Ludwig-Maximilians University of Munich (Germany) with a focus on the field of interactive technology. Her research group explores design factors and relevant psychological mechanisms in the context of technology usage in different fields, e.g., social media, digital collaboration, companion technologies, social robots. Since 2007 she is engaged in research on user experience and consumer experience in the field of interactive products. Current research topics focus on the negative side effects of technology use on happiness and wellbeing (“digital depression”), the psychological effects of social media (e.g., selfie-paradox) as well as interaction design from a psychological perspective (e.g., aesthetics of interaction, psychological needs approach). Sarah Diefenbach developed several methods for user experience design and evaluation (e.g., interaction vocabulary) which are widely applied in research and practice. Her research is internationally published and recognized, including more than 100 articles in books, journals, and conference proceedings.

Three studies conducted within the FAI by students for their bachelor theses also addressed the situation of employees in a teleworking environment. First, Braune (2021) conducted a survey study with 69 employees, comparing their cybersecurity intentions when 1) working in a telework set up and 2) working on site at their organization. She did not identify any notable differences in cybersecurity intentions between on site and telework situations.

Second, Dressler (2021) conducted interviews with 20 employees who had to switch to telework in the wake of the COVID-19 pandemic in 2020. Her primary focus was on whether and how participants employed impression management tactics in this digital setup. In this context, impression management refers to the attempt of a person to control the impression they leave on other people (Schlenker 1980; Goffman 1959). Moreover, she explored how the communication changed when her participants were forced to rely on telework. Hence, we will elaborate in more depth on her study design and results in the following section. The third study (Weichert 2022) built on Dressler’s results and investigated, whether a video-call with the camera on or off affected attentiveness and concentration performance in a dual task.

### Method

In total, interviews were conducted with 20 interviewees (8 identified as female, 12 as male), aged between 25 and 65 years (M = 41.90; SD = 11.8). Seven participants worked in the IT industry in project management, programming, or consulting, two of the participants were still studying, another two were working in office management, two were self-employed and two designers. The remaining five participants had a professional background in facility planning, government, consulting, process management, and finance.

The interviews were conducted using a video-call software and lasted between 28 and 66 min (M = 40 min). Prior to the interview calls, all participants had been asked to sign an informed consent sheet via email. The interviews consisted of seven main parts:

1. Welcome: First, the interview participants were welcomed and thanked for their participation.
2. Communication: Second, they were asked about their communication with their environment (professional or private) and how this communication has changed over the time while they were working remotely.
3. Videoconferencing: The following questions were related to participants’ behaviour in videoconferences. There were no direct questions about self-representation behaviour, but if the answers tended in that direction, a more detailed explanation was requested. In addition, questions were asked about the use of virtual background pictures—whether they used such background pictures themselves or their communication partners. Participants were also asked about real-life backgrounds, especially whether they had picked their backgrounds on purpose and whether they monitored others’ real-life backgrounds with interest.
4. Visual appearance: The questions here related to clothing and personal hygiene in the home office. Participants were asked if anything had changed in the home office compared to working on site. Impression-managements behaviour was not directly asked about here either but encouraged if related answers were given.
5. Facial expressions and gestures: The following questions referred to facial expressions and gestures in video conferences. It was asked, how important these were perceived to be, both by the participants themselves and by their communication partners.
6. Self-perception: The participants were asked whether they think about how they appear in video conferences and whether it is important to them to be perceived positively.
7. Finally, participants were asked to provide some demographic details. Then, they were thanked again for their participation and provided with the opportunity to ask questions.

The interviews were audio-recorded and transcribed for analysis. The data was analysed using thematic analysis (Braun and Clarke 2006). After reading the transcripts multiple times, the author identified themes by coding the transcripts on the sentence level, going back and forth multiple times to further refine the codebook. Successively, a codebook emerged with four main categories, 14 subcategories and 70 codes. We focus here on the results regarding the first research question pursued by Dressler (2021): How has the communication changed by the shift from working on site to teleworking? Dressler identified four themes: (1) limited communication, (2) spontaneity, (3) increased stress, and (4) advantageous aspects.

### Results

The results regarding how communication has changed by shifting to teleworking are described below.

#### Limited communication

Half of the participants (*N* = 10) reported that communication with their colleagues was strictly limited to business-related topics: “Communication is mostly really work-related and we don’t take much time to talk about other stuff beyond that” (P20). Eight participants indicated that small talk no longer takes place: “You don’t go into a meeting room and chat a bit. Or take a break and have a coffee.” (P09). A quarter of the participants (*N* = 5) said that communication has now shifted more to writing and less exchange takes place in oral form: “So with us now already less is spoken, but more is written” (P03) and “Well, it has actually shifted more to writing” (P13). Several participants (*N* = 7) also described that they no longer had any personal contact with their colleagues: “Instead of direct contact across the desk … now exclusively via video conferences or telephone calls or e‑mail” (P19). Several (*N* = 8) participants also reported a very limited circle of people with whom communication still takes place: “You just call only the people you need and don’t chat now with people you may have seen twice” (P06) and “Now you really only have contact with the closer work colleagues” (P02).

#### Spontaneity

Some participants (*N* = 6) complained about the loss of spontaneity due to working remotely: “Well, what has definitely changed is this less spontaneity” (P01) or “You just don’t meet by chance at the coffee machine anymore” (P05). Several (*N* = 5) also mentioned that spontaneous queries are no longer possible: “Communication is limited to what you ask or write … if you were to speak, I could ask again right away or there would be another query …” (P09). Another five participants described that more planning has become necessary: “That is, you bundle up things that are on your mind that you want to discuss sometime and then you address them all … whereas before you might have turned around three times in half an hour and briefly asked a question.” (P20) or “That’s actually the main difference from my point of view, that I have to do everything much more purposefully, much more planned, more deliberately, more consciously. What used to happen incidentally.” (P05). Eight of the participants also mentioned that communication itself takes more time, “because it sometimes takes longer to clarify things and then you wait in the chat until the other person answers” (P17).

#### Increased stress

A few participants (*N* = 3) stated that their stress level had increased: “I also have the feeling that I am always more stressed in group work” (P01). Five participants explained that technical problems also increased their stress level: “It was also … stressful when my own Internet connection didn’t cooperate” (P01) and “The technology is still a limitation, because usually only one person comes through who talks and even if you try to interrupt him, it usually doesn’t get through to the person who is talking.” (P17).

#### Advantageous aspects

A quarter of the participants (*N* = 5) indicated that they now had more control over interruptions from colleagues: “Because you have more control over who you talk to and when. … As opposed to the office, where you were available at any time—if someone wanted to talk to you.” (P19). One participant also described that collaboration was often easier now: “Discussing things that we show each other on our screens is easier than it used to be because they can both sit centrally in front of their screens … whereas if I go over to my colleague’s desk and look over his shoulder, it’s been a suboptimal situation in the office. … Screen sharing is beneficial for a lot of things.” (P20)

The study results further indicate that self-presentation in the form of elegant work attire and a well-groomed appearance as well as active body language also plays a major role when working remotely and that a reduction in these forms of self-presentation can only be observed among a few participants. Most participants clearly stated their desire to be perceived positively by their interlocutors, even when communicating via digital media. Half of the participants were neutral about the use of a virtual background by their communication partners. Still, a few participants reported that this background change aroused a certain curiosity in them and they wondered what exactly was supposed to be hidden. An increased sense of distance as a consequence of hiding one’s real background was also mentioned in one interview (Dressler 2021).

Finally, Weichert (2022) build on these results to conduct a group experiment following a within-subject design. In seven sessions with a total of 35 participants (of whom 34 completed the study questionnaire), Weichert (2022) investigated whether participants in a video-call with the camera on or off showed a higher attentiveness and concentration performance using a dual task approach. The results did not indicate an effect of having the camera on or off on the participants’ attentiveness or concentration. However, the study may have been underpowered due to the small sample size and further research is needed to explore this topic in more depth (Weichert 2022).

## Need fulfillment through interaction with technology

Lara Christoforakos and Sarah Diefenbach address a topic related to that described in the previous section in their article: They conducted a qualitative in-depth interview study to explore how interaction with technology can fulfill social needs and how anthropomorphism relates to that need fulfillment.

Lara Christoforakos is a PhD student, research and teaching assistant at the Chair of Economic and Organisational Psychology at Ludwig-Maximilians-University Munich since 2017. From 2015 to 2017, she was a student research assistant at that Chair. From 2014 to 2017, she completed her Master’s programme in Economic, Organizational and Applied Social Psychology (Wirtschafts‑, Organisations- und Sozialpsychologie; Master of Science) at Ludwig-Maximilians-University Munich, and from 2010 to 2014 her Bachelor’s programme in Psychology (Bachelor of Science) at Ludwig-Maximilians-University Munich.

In their article, Christoforakos and Diefenbach focus on the fulfillment of social needs. As described in the second section, we also conducted several studies that aimed to shine light on how psychological needs, including but not limited to social needs, can be addressed by users’ interaction with different technologies (e.g., Zimmermann and Gerber 2020a). In an additional survey study not described above, Gerber (2020) asked 246 participants (of whom 112–46%—identified as female and 134–54%—as male; M = 36.07 years old, SD = 11.46; min = 18, max = 70) to indicate on a 5-Point-Likert scale (1 = strongly disagree; 5 = strongly agree) whether they felt that the ten psychological needs described by Sheldon et al. (2001) were fulfilled through their use of various technologies. Need fulfillment was assessed using the Needs Scale developed by Diefenbach et al. (2014).

**Fig. 3:**
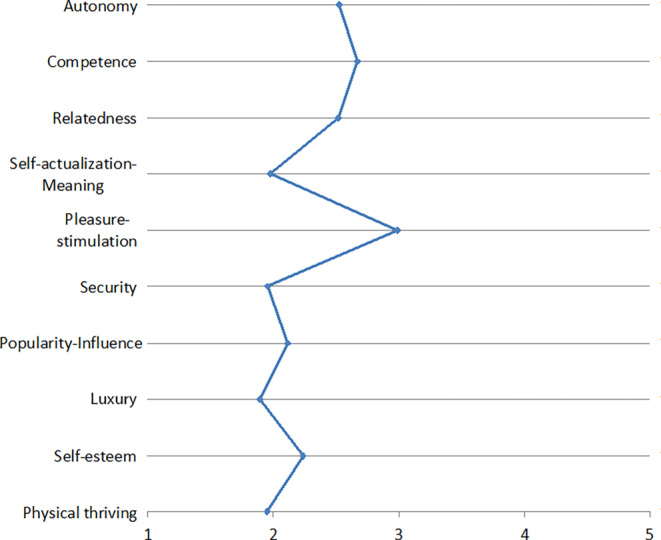
Needs associated with using game consoles Bedürfnisse, die mit der Nutzung von Spielekonsolen verbunden werden

**Fig. 4:**
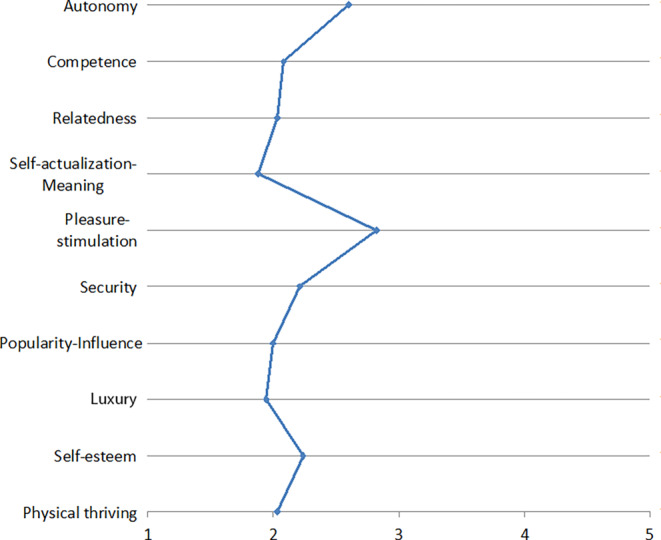
Needs associated with using smart TVs Bedürfnisse, die mit der Nutzung von smarten Fernsehern verbunden werden

**Fig. 5:**
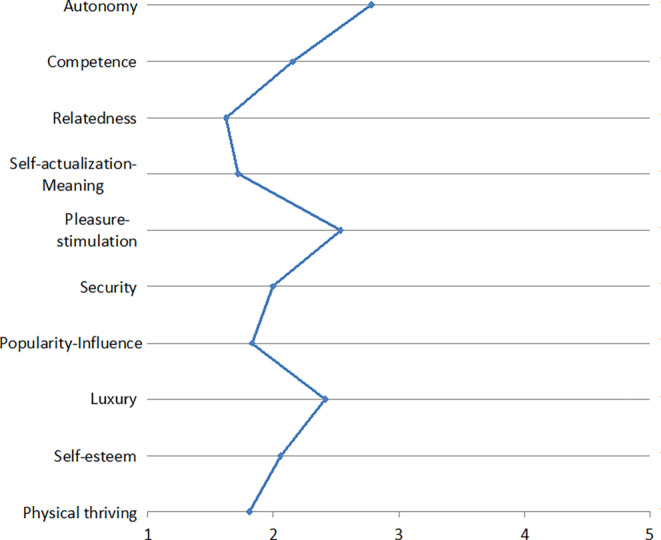
Needs associated with using E‑Commerce services Bedürfnisse, die mit der Nutzung von E‑Commerce-Diensten verbunden werden

**Fig. 6:**
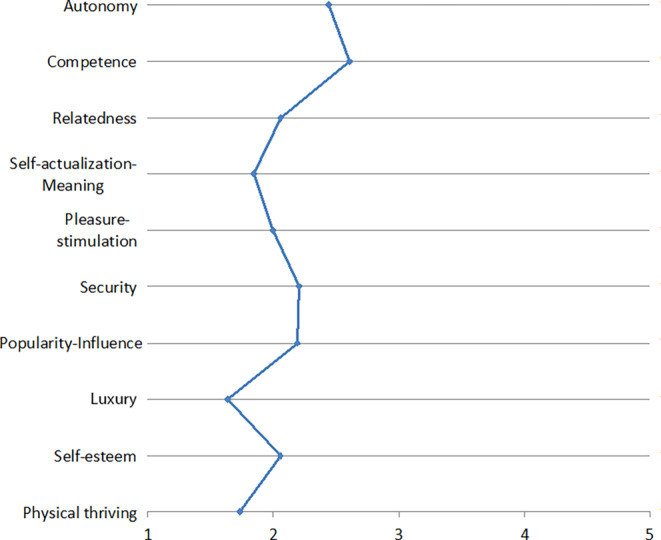
Needs associated with using cloud services Bedürfnisse, die mit der Nutzung von Cloud-Diensten verbunden werden

**Fig. 7:**
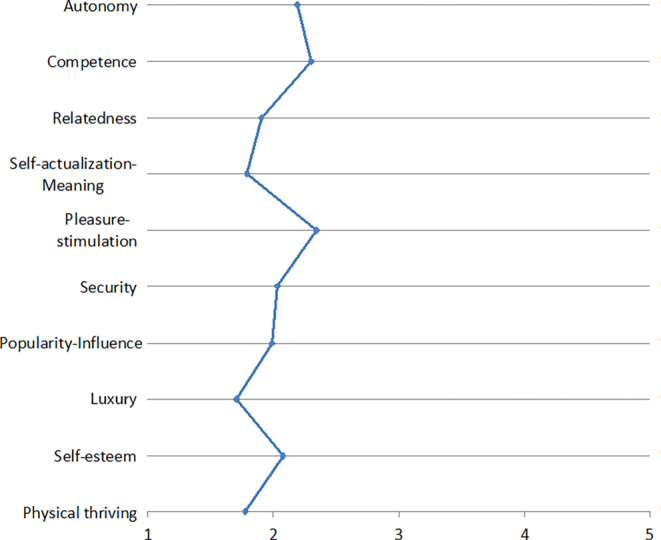
Needs associated with using digital assistants Bedürfnisse, die mit der Nutzung von digitalen Assistenten verbunden werden

The resulting need profiles are depicted in Figs. 1, 2, 3, 4, 5, 6 and 7. According to the results, the social need, in this case relatedness to others, was mostly addressed by interacting with messengers and social networks. Still, in contrast to the study described by Christoforakos and Diefenbach, the participants in Gerber’s (2020) study did not report to fulfill this need solely based on the interaction with the technology. In this example, it can rather be assumed that the technology serves as a mediator with the help of which communication or interaction with other people takes place. Therefore, the fulfillment of needs cannot be directly attributed to the use of the respective technology as in the case of Christoforakos and Diefenbach.

A need that was frequently stated as being fulfilled more than average is stimulation. This was associated with the use of social networks, game consoles, smart TVs, and E‑Commerce services. The latter was also reported to fulfill the needs autonomy and luxury by the study participants. Autonomy was further indicated to be addressed by interacting with smart TVs and cloud services, which are further associated with the feeling of competence. Digital assistants, a technology that was also mentioned by several participants in the study described by Christoforakos and Diefenbach, were found to mainly address stimulation, competence, and autonomy, although none of these needs was reported to be met even moderately by interacting with digital assistants. The results of Christoforakos and Diefenbach can provide potential reasons for this comparatively poor performance of digital assistants, which lie in the currently not yet fully developed design of these technologies and also lead to the fact that these technologies, developed for the simulation of a human interaction, are perceived as rather less anthropomorphic.

## Industrial human-interface design

Mucha, Robert, Breitschwerdt and Fellmann describe in their article the importance of usability for a clinical decision support system (CDSS) and provide a case study to illustrate how they used codesign to produce usable software in a real-world context. Henrik Mucha is a research associate at the Fraunhofer Institute of Optronics, System Technologies and Image Exploitation (IOSB) in Karlsruhe and a lecturer in Design Methods and Interaction Design at the Rhine-Waal University of Applied Sciences. Currently, he is doing his PhD on human-centered and participatory design of Decision Support Systems (DSS) with a focus on medical issues and applications. Sebastian Robert is a professor for IT and data management in healthcare at the Rosenheim University of Applied Sciences. He investigated on how human-centered design methods can be used to develop decision support systems for molecular tumor boards. Rüdiger Breitschwerdt is professor in the field of health IT at the Wilhelm Büchner University of Applied Sciences in Darmstadt. His research focuses on IT-based prevention including information systems for healthcare facilities. Michael Fellmann holds a Junior Professorship in Business Informatics with a focus on “Business Information Systems” at the Faculty of Computer Science and Electrical Engineering at the University of Rostock. The research interests of Michael Fellmann are Business Process Management, Mobile and Wearable Systems and Service Science.

Optimal human interface design is one of the main research areas of the FAI working group. Within the framework of third-party projects, bachelor- and master theses as well as study projects, the terms of usability and user experience have been analysed in different working environments. One of the latest projects in this area is WaReIp (Water-Reuse in Industrial Parks www.wareip.de), a joint project funded by the Federal Ministry of Education and Research (BMBF) within the framework of the funding measure “Future-oriented technologies and concepts for increasing water availability through water reuse and desalination” (WAVE). The aim of the research project was the reuse of wastewater generated within an industrial park by using different treatment stages. The water flows had to be controlled and monitored by a specialized staff operating a new control system made up of several surveillance monitors and a control panel. The role of the psychologists involved in the project was to investigate the design of the working conditions of the control room staff to make sure that they can make the right decisions under high stress and in complex situations.

Therefore, a dashboard prototype was designed and tested in terms of usability, understanding and intuitive operation. The purpose of the dashboard was to support the operators in their daily tasks, e.g., process monitoring, diagnosis of certain water parameters and interventions in case of malfunctions. Some of the key parameters on the dashboard included temperature, pH value, wind speed, and pressure of the water flows.

**Fig. 8:**
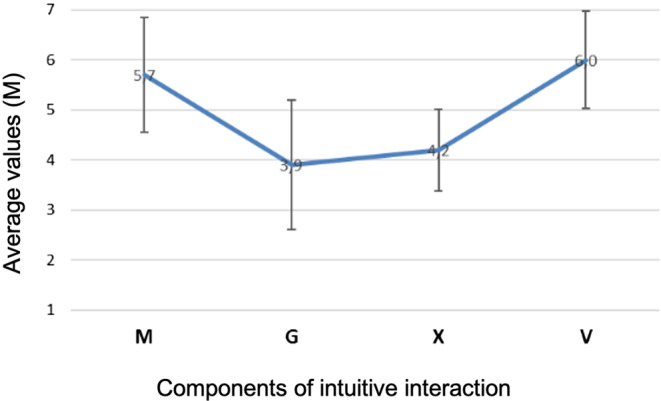
Average values of the four components of the INTUI questionnaire: *M* (Effortlessness), *G* (Gut Feeling), *X* (Magical Experience), *V* (Verbalizability) Durchschnittswerte der vier Komponenten des INTUI-Fragebogens: *M* (Mühelosigkeit), *G* (Bauchgefühl), *X* (Magische Erfahrung), *V* (Verbalisierungsfähigkeit)

The usability of the dashboard was tested with both unexperienced users and usability experts by using both qualitative and quantitative methods. A total of 18 unexperienced users interacted with the dashboard by solving different tasks, e.g. to identify the actual zone, the values for the different parameters, temperature, wind speed or to interpret the interface design and colour interpretation. The usability method chosen for this interaction is called “Think-Aloud” and implies, that the subjects speak out their thoughts during the interaction with the dashboard and an investigator writes these thoughts down and uses them for the overall assessment. After the interaction, the subjects assessed with the INTUI questionnaire by Ulrich und Diefenbach (2010), how intuitive they perceived the interaction with the dashboard to be (see Fig. 8).

In addition to the Think-Aloud Method, a heuristic evaluation (Berliner Kompetenzzentrum für Usability-Maßnahmen 2016) with four experts was also conducted. The Berlin Competence Center for Usability Measures (2010) recommends a heuristic evaluation as a supplement to a usability study because it is economic, many usability problems can be uncovered, and heuristic evaluation can take place in all phases of product development. Provided by the German Institute for Standardization (Deutsches Institut für Normung 2017), the following principles of the ISO 9241—112:2015 Ergonomics of Human-System Interaction—Part 112: Principles for the presentation of information (2015) were selected as heuristics and assessed on a scale from 1 (cosmetic problem, only eliminate if enough time) to 4 (usability disaster, absolutely must be eliminated): detectability, freedom from distraction, discriminability, unambiguous interpretability, conciseness and consistency. In order to get a precise assessment, the dashboard was divided into three components which were assessed individually: header including graphic, process monitor and process visualization. For all three components the experts were able to identify cosmetic, small and big usability issues. The findings were reported to the project manager in order to be discussed and implemented. When designing software interfaces, usability is crucial for performing the work tasks with effectiveness, efficiency and satisfaction and is a quality criterion in every working environment.

## Human factors in aviation research

The article of Christoph Santel “A time machine for accident investigators: Reconstructing the likelihood of detecting traffic during a midair collision using simulator-based experiments with flight crew” focuses on the investigation of an aircraft accident in which two light aircrafts collided near Frankfurt in 2012 and ended with eight human victims. The aim of the investigation was to collect insights that may help preventing future collisions.

Christoph Santel is a Senior Research and Development Engineer at Garrecht Avionik GmbH in Walldorf where he supports the development operation for aviation electronics. He has a technical expertise in the design of human-machine interfaces and avionics systems and was accredited by the European Association for Aviation Psychology as a Human Factors Specialist. During his mechanical engineering studies at RWTH Aachen University and while working as research associate at the Institute of Flight Systems and Control Engineering (Chair of Uwe Klingauf) at the Technical University of Darmstadt, Christoph Santel devoted himself to aerospace engineering. He earned his doctorate with the development and usability testing of collision alerting systems in general aviation, Joachim Vogt being his co-referee.

Working in a cockpit environment in a safe manner requires among other qualities also vigilance, i.e., an “individual’s ability to pay close and continuous attention to a field of stimulation for a period of time, watchful for any particular changing circumstances” (Skybrary o.D.). Together with the Institute of Flight Systems and Automatic Control from TU Darmstadt and industry partners, psychologists from the Work and Engineering Psychology Research Group (FAI) develop a virtual cockpit assistant (VCA) for the pilots and investigate how artificial intelligence can be made usable in cockpits. Different use cases have been identified together with experienced pilots and prioritized according to applicability and importance. Special attention is paid to the cooperation between humans and the developed intelligent systems. To this end, interfaces will be designed that promote an effective cooperation between humans and autonomous systems. In particular, the impact of safety-critical environments such as the cockpit of an aircraft on the design of the interfaces must be investigated and taken into account.

The virtual cockpit assistant will not be considered a tool controlled by the human, but seen as an (almost) equally placed team member (Demir et al. 2017; Schaefer et al. 2016). This approach is part of the Human-Autonomy Teaming (HAT) and describes the collaboration between humans and highly automated agents that work together towards a common goal. The VCA will be tested in several flight simulator studies, examining issues related to functionality, usability, user experience, and acceptance.

The project is part of the Federal Aeronautical Research Programme (Luftfahrtforschungsprogramm LuFo VI) and supported by the Federal Ministry for Economic Affairs and Climate Action.

More information about the aviation research within FAI can be taken from the following publications:

- Santel, C, Gerber, P, Mehringskötter, S, Schochlow, V, Vogt, J, Klingauf, U (2014) How glider pilots misread the FLARM collision alerting display: A laboratory study. In: Aviation Psychology and Applied Human Factors, 4 (2), S 86–97
- Santel, C, Gerber, P, Mehringskötter, S, Schochlow, V, Vogt, J, Klingauf, U (2013) Verkehr auf 10 Uhr, hoch? Wie Segelflugpiloten die Anzeige des FLARM-Kollisionswarngerätes falsch interpretieren. 37. Symposium für Segelflugzeugentwicklung, Braunschweig
- Schochlow, V, Santel, C, Weber, C, Vogt, J, Klingauf, U, Grandt, M, Schmerwitz, S (Hrsg.) (2012) Kollisionsvermeidung im Luftsport: Eine experimentelle Studie der Mensch-Maschine-Schnittstelle eines populären Kollisionswarnsystems in der allgemeinen Luftfahrt. 54. Fachausschusssitzung Anthropotechnik: Fortschrittliche Anzeigesysteme für die Fahrzeug- und Prozessführung, Koblenz, S 157–173
- Wenzel, M, Sprenger, E, Pasnicu, O, Staudt, J, Ellenrieder, N (2022) Virtual Flight Deck Crew Assistance utilizing Artifical Intelligence Methods to interpret NOTAMS—A User Acceptance Study. Deutscher Luft- und Raumfahrtkongress, Dresden, Germany

## Human-centered design in automotive research

In their paper “Towards a guide for developers and novice researchers on human-centered design of the take-over request—Combining User Experience and Human Factors”, Lindner and Stoll illustrate a human-centered design process for interdisciplinary development and research teams, using an exemplary application in the field of highly automated driving. They suggest combining methods from the field of human factors as well as methods from the field of user experience, which will lead to increasing safety and comfort, as well as taking human needs into account. This approach can contribute to the development and design of all levels of automation.

Prof. Dr. Alisa Lindner was born in 1989 and has been a professor for User Experience Design in Autonomous Driving at Coburg University of Applied Sciences since 2021. Previously, she worked on improving user-centricity of the engineering process at Robert Bosch Automotive Steering GmbH. She studied work and engineering psychology at the TU Darmstadt and did her PhD in cooperation with Daimler AG in the field of subjective evaluation of steering feel on heavy commercial vehicles.

Tanja Stoll, born in 1988, is a research associate in the field of Human Factors Psychology at the Zurich University of Applied Sciences. She studied Psychology and Human Factors Engineering and is currently completing her doctoral thesis on cooperatively interacting vehicles at the Department of Human Factors at Ulm University.

On the one hand, the mobility market is characterized by the increasing complexity and number of variants of products as well as the short-lived nature of technological innovations (Tomforde 2005; Braess 2011). On the other hand, the mobility market is confronted with growing customer demands for quality, safety, value, functionality, and image. In the volume market in particular, customers have low price expectations due to high market saturation. For the premium segment, substance and authenticity are drivers of success (Tomforde 2005). A strong brand code can be conveyed, among other things, by a distinctive “driving experience” as a differentiating and marketing feature. Expectations for the car of the future and reasons for owning a car also reflect this (Statista 2014; Statista 2018). Alongside “safety,” “price,” “environmental compatibility” and “suitability for everyday use,” the characteristics “comfort” and “driving pleasure” represent the most important expectations of “cars of the future”.

FAI has conducted research on experiencing joy and convenience in highly automated driving and in Human-centered design of Smart Car Interfaces. Further information can be taken from the following publications:

- Menig, A (2018, June). Joy and convenience in highly automated driving. In B Schick, & C Seidler, Driving fun, comfort and stress in autonomous driving—a human centered approach (workshop). Research presentation at the 15th International Conference on Intelligent Autonomous Systems, Baden-Baden, Germany
- Pasnicu, O, Zimmermann, V, Gerber, N & Cardoso, S, (2022). Autonomous Driving—Analysing the Impact of Resilience Engineering Features in Smart Car Interfaces. In: M Mühlhäuser, C Reuter, B Pfleging, T Kosch, A Matviienko, K S Gerling, W Heuten, T Döring, F Müller & H Schmitz (Hrsg.), Mensch und Computer 2022 – Tagungsband. New York: ACM. (pp. 537–541). 10.1145/3543758.3547573

## Human perceptual aspects in the design of products

In their paper “Towards intelligent illumination systems: from the basics of light science to its application”, Zandi and Khanh give an insight into the interdisciplinary research field of Human Centric Lightning. The new generation of multi-channel LED luminaires offer the possibility to display the light spectrum in a flexible and adaptive way. Human-centric design of intelligent lighting systems should include both, the sensitivity of the human visual system and individual visual preferences on lightning, as well effects of light on human melatonin suppression. Due to the need to save energy, it is of high interest to achieve the best possible lighting level with the lowest possible energy usage. According to Zandi and Khanh, office lighting is an important application.

Babak Zandi received his M.Sc. and doctoral degree in electrical engineering from the Technical University of Darmstadt. Currently, he is working as a research group leader at the Laboratory of Adaptive Lighting Systems and Visual Processing (Technical University of Darmstadt). Further, he serves as an editorial board member at Scientific Reports. His scientific interests include the integration of neuronal networks in heuristic optimization procedures and the time- and spectral-dependent modelling of the human pupil light response. He also deals with various psychophysical and statistical methods to carry out and analyse investigations in automotive lighting.

Tran Quoc Khanh is professor for Adaptive Lighting Systems and Visual Processing at the Technical University of Darmstadt (Germany). He received his Dr. Ing. at the Faculty for Physics and Electronic Devices from the Technical University of Ilmenau (Germany). From 1990 to 2006 he worked as a developing engineer and project manager for radiometric, colorimetric and photometric systems (in Munich and Berlin, Germany), and for digital cinema cameras, laser recorder, laser scanner and LED luminaires (in Munich, Germany). He habilitated on mesopic vision, colour image processing and colour appearance.

In car development, especially in the premium segment, not only vision, but also touch, feel, Cochlear sense, and proprioception are important. The optimization of drivability, i.e. the customer-perceivable response of a passenger car to the driver’s acceleration request via the accelerator pedal, plays a major role (Müller et al. 2012). FAI is part of joint projects with industry and the Institute for Mechatronic Systems (IMS) at TU Darmstadt to investigate human perceptual and psychological aspects of longitudinal dynamic driving manoeuvres in different powertrain configurations. The studies are conducted both in a dynamic driving simulator and in a real vehicle. In stress-strain studies with secondary tasks, physiological parameters, like heart rate and skin conductance response, are also recorded.

Further information can be taken from the following publications:

- Erler, P, Menig, A, Uphaus, F, Malonga Makosi, C, Rinderknecht, S, & Vogt, J (2018, July). Investigating the perception of powertrain shuffle with a longitudinal dynamic driving simulator. In International Conference on Advanced Intelligent Mechatronics (IEEE, ASME), Auckland, New Zealand
- Menig, A, Breitenbach, G, & Vogt, J (2017, September). Praxisrelevante Erkenntnisse zur Erhebung der physiologischen Beanspruchung in Fahrsimulatorstudien – Einfluss des psychischen Ausgangszustands auf die Baseline-Messung. Posterbeitrag zur 10. Tagung der Fachgruppe Arbeits‑, Organisations- und Wirtschaftspsychologie (DGPS), Dresden, Germany
- Kraft, E, Pasnicu, O, Vogt, J, Rinderknecht, S (2020). Influence of engine sound on the evaluation of simulator driving (Posterbeitrag). In Proceedings of the Driving Simulation and Virtual Reality Conference & Exhibition: New trends in Driving Simulation & VR. Science and Technology (pp. 201–202)
